# Primary Pulmonary Malignant Melanoma: Report of an Important Entity and Literature Review

**DOI:** 10.1155/2017/8654326

**Published:** 2017-03-02

**Authors:** Christos Kyriakopoulos, George Zarkavelis, Artemis Andrianopoulou, Alexandra Papoudou-Bai, Dimitrios Stefanou, Stergios Boussios, George Pentheroudakis

**Affiliations:** ^1^Department of Pulmonary Medicine, Medical School, University of Ioannina, Stavros Niarchos Avenue, 45110 Ioannina, Greece; ^2^Department of Medical Oncology, Medical School, University of Ioannina, Stavros Niarchos Avenue, 45110 Ioannina, Greece; ^3^Society for Study of the Clonal Heterogeneity of Neoplasia, Ioannina, Greece; ^4^Department of Radiology, Medical School, University of Ioannina, Stavros Niarchos Avenue, 45110 Ioannina, Greece; ^5^Department of Pathology, Medical School, University of Ioannina, Stavros Niarchos Avenue, 45110 Ioannina, Greece

## Abstract

Malignant melanoma involving the respiratory tract is nearly always metastatic in origin, and primary tumors are extremely rare. Published data on primary pulmonary malignant melanomas are limited. Up to now 40 relevant cases have been reported in the English literature. Herein, we report a case of a 56-year-old female patient who presented with intracranial metastases due to primary pulmonary melanoma. She underwent bronchoscopy and died 5 months after the initial diagnosis despite the administered biochemotherapy and subsequent immunotherapy. To establish the diagnosis of primary pulmonary malignant melanoma, any extrapulmonary origin was excluded by detailed examination and radiographic imaging. Moreover, an extensive review of the literature regarding this rare entity has been performed.

## 1. Introduction

Worldwide, approximately 160,000 new cases of melanoma are diagnosed each year, and about 41,000 melanoma-related deaths occur annually [1, 2]. Melanoma is a malignant neoplasm of melanocytes, and more than 90% of the reported cases are cutaneous in origin. Although malignant melanoma mainly occurs on the skin, it has also been described in other mucosal sites and organs, including the oral cavity, paranasal sinuses, esophagus, larynx, vagina, anorectal region, and liver [2, 3]. Approximately 5–10% of patients with metastatic melanoma have a primary melanoma of unknown origin [2, 4]. Primary malignant melanoma of the lung is an extremely rare nonepithelial neoplasm that accounts for only 0.01% of all primary lung tumors [1, 5].

To date, 40 cases have been reported in the English literature [6–41]. Patients' characteristics and demographics are depicted in Table 1. The mean age at diagnosis is 59.1 years (range 29 to 90) with male prevalence. Melanomas involving the lung are almost always metastatic, and it is extremely rare to find a true primary lesion. Metastasis from an occult primary lesion must be excluded by proposed criteria [3, 4, 31].

## 2. Case Presentation

**Figure 1:**
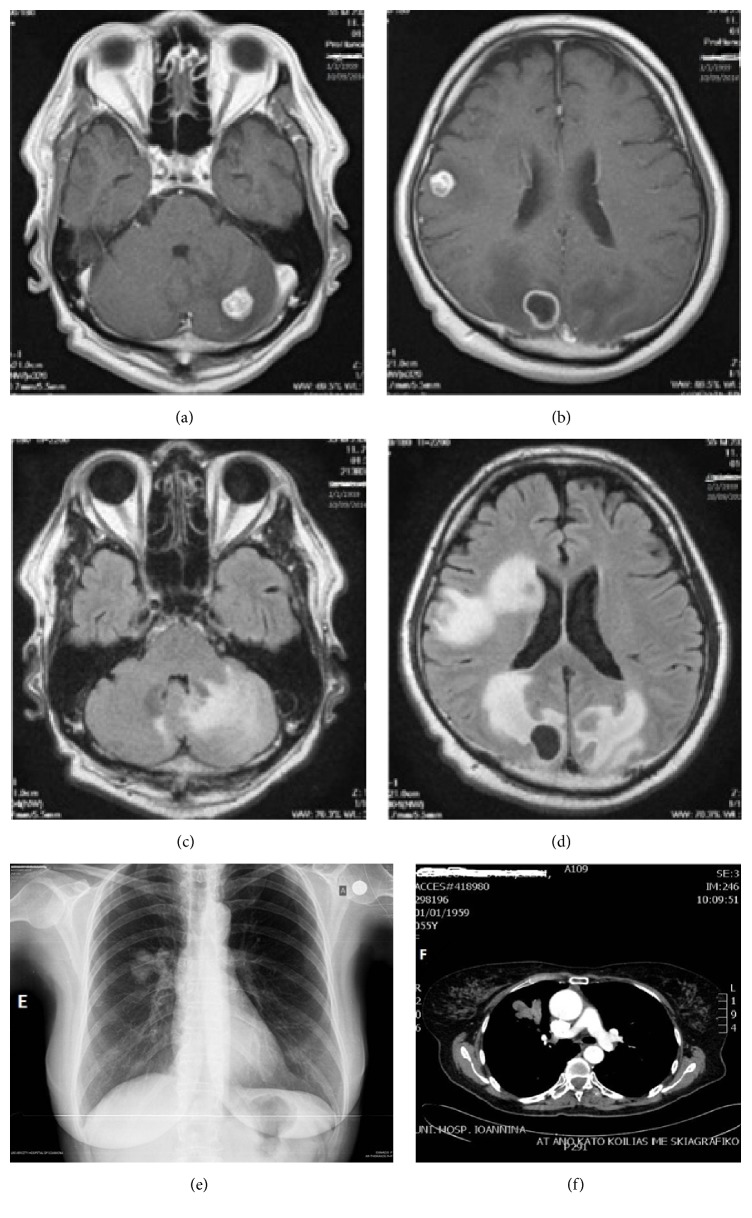
((a) and (b)) Postgadolinium T1-weighted MR images demonstrate multiple enhancing nodular lesions throughout both cerebral and cerebellar hemispheres, several of which have an unusual lamellated pattern of enhancement. ((c) and (d)) T2-weighted and FLAIR MR images with paired postgadolinium T1-weighted images demonstrate significant vasogenic edema associated with several of these lesions. (e) Chest X-ray showing tumor in the anterior segment of right upper lobe. (f) Chest CT showing tumor in the anterior segment of right upper lobe with ill-defined shape and right hilum lymph nodes.

**Figure 2:**
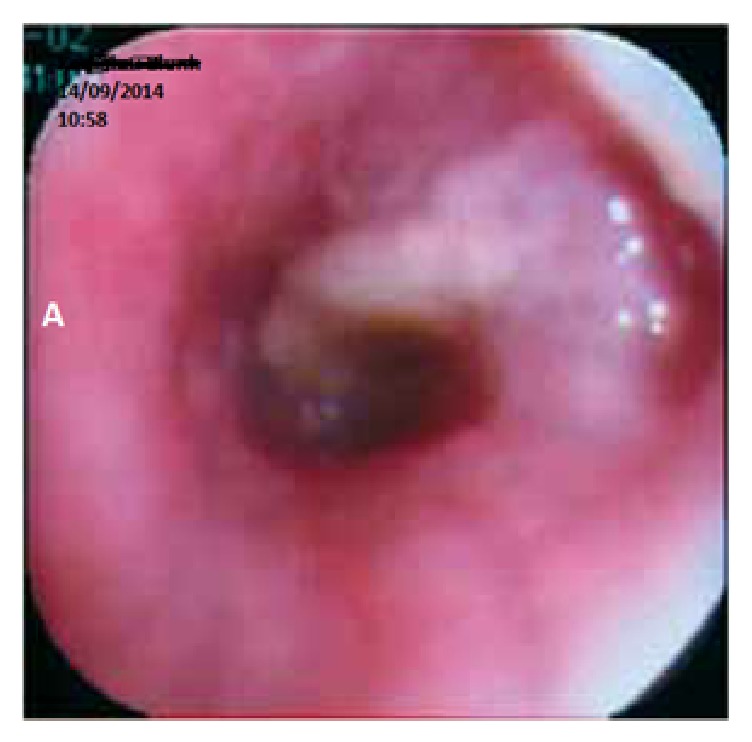
Bronchoscopic examination showed a bulging lesion in the bronchus of the anterior segment of the right upper lobe.

**Figure 3:**
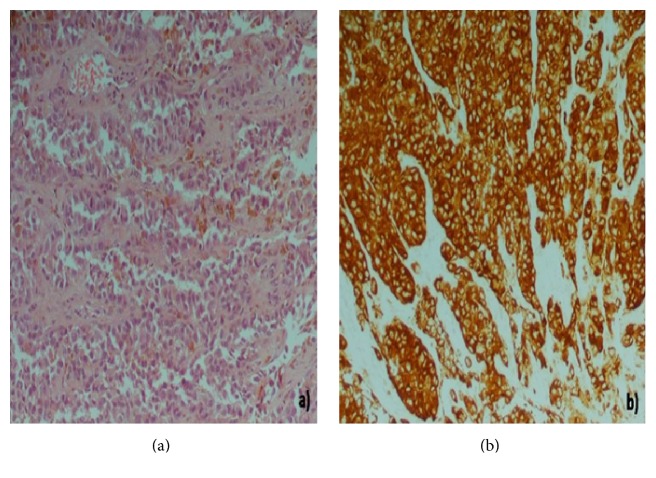
(a) Bronchial biopsy with infiltration by atypical melanocytes containing melanin pigmentation (*H&E, magnification *×*200*). (b) Immunohistochemical expression of Melan A in neoplastic cells (*DAB, magnification ×200*).

A 56-year-old female without comorbidities and a 50-pack-year smoking history was referred to the emergency department with a severe right frontal headache. The onset was abrupt and the preceding symptoms and signs included nausea and ataxic gait. Neither cranial nerve abnormalities nor focal limb weakness was spared. Initial laboratory values were within the reference range. The patient underwent a magnetic resonance imaging (MRI) which revealed multiple nodular lesions throughout both cerebral and cerebellar hemispheres with an unusual lamellated pattern of enhancement. The demonstrated significant vasogenic edema did not led to midline-shift (Figures 1(a)–1(d)). Chest X-ray detected opacity with a lobulated contour in the right upper lobe (Figure 1(e)). A contrast-enhanced chest computed tomography (CT) showed an ill-defined mass in the anterior segment of the right upper lobe and right hilar lymphadenopathy (Figure 1(f)). No additional abnormalities were observed on subsequent detailed metastatic work-up including abdominal CT and whole body bone scintigraphy. Due to the neurological symptoms, the patient underwent whole-brain radiotherapy (WBRT). Fractionation of 3 Gy per day was used, reaching a total dose of 30 Gy in two weeks; in addition, the patient received intravenously dexamethasone during WBRT. The performed bronchoscopy detected a bulging lesion located at the membranous portion of bronchus of the anterior segment of the right upper lobe. The lesion did not appear to be covered with normal bronchial mucosa. Subsequently, a biopsy was performed (Figure 2), and the histopathology showed infiltration by atypical melanocytes containing melanin pigmentation (Figures 3(a) and 3(b)). Positive immunohistochemical staining of MART-1, S-100 protein, and HMB-45 and lack of expression of cytokeratin (CK), epithelial membrane antigen (EMA), synaptophysin (Syn), and high molecular weight cytokeratin confirmed the diagnosis of pulmonary melanoma. Molecular profiling was conducted which failed to detect any mutations.

To rule out the diagnosis of melanoma metastasis from a primary site, an extensive skin and fundoscopic examination was carried out. Endoscopy of both upper and lower gastrointestinal tract as well as cystoscopy did not detect any additional abnormalities. Indeed, a panendoscopy of the upper airway failed to demonstrate extrapulmonary disease. Based upon the histological characteristics, immunohistochemical features, and clinical data, the diagnosis of primary pulmonary malignant melanoma was established.

Surgical resection of the lung mass was not indicated due to the brain metastases. The completion of WBRT resulted in transient remission of the neurological symptoms and the patient was switched to dacarbazine (DTIC) in combination with recombinant interferon alpha-2a (rIFN-*α*-2a). Due to unacceptable toxicity rIFN-*α*-2a was discontinued three weeks after being administered.

The treatment did not provide any clinical benefit; instead patient's condition deteriorated and ipilimumab was added to DTIC. The evaluation of response was planned to be performed after four cycles of the administered combination therapy. Indeed, the patient was admitted to the hospital with progressive exertional dyspnoea and persistent chest tightness. She died five months after the initial diagnosis. Massive pulmonary embolism was determined as the cause of death in the performed autopsy.

## 3. Discussion

Published data on primary pulmonary malignant melanoma are limited. The performed systematic review confirms that only 40 cases appeared in the literature since 1916 (Table 1) [6–41]. Median age of presentation was 59.1 years (range, 29 to 90). The gender distribution for the entire population was 21 males to 19 females. Therapeutic management is a great challenge, and clinical trials are clearly warranted to evaluate the efficacy of local and systemic adjuvant treatments in decreasing recurrence and improving survival. Surgical resection was the primary treatment (27 out of the 40 reported patients, 67.5%) while adjuvant systemic treatment was delivered just to 19 patients (47,5%). In addition, palliative radiotherapy to distant sites had been administered in 4 patients (10%). In general, it seems that most patients (19 out of 40) were diagnosed with metastatic disease (47,5%). The sites of metastatic involvement included the contralateral lung, liver, brain, bones, and pericardium. The outcome was very dismal and the majority (26 out of 40 patients) survived less than 18 months (65%).

Primary pulmonary malignant melanoma resembles that of the skin or mucosa. A diagnosis of primary malignant melanoma of the lung is based on clinical and pathological criteria [4, 26, 42]. The clinical criteria include absence of either history suggestive of a previous melanoma or demonstrable melanoma outside the thorax at the time of surgery. Furthermore, the presence of a solitary lung mass or nodule is required. The established pathological criteria incorporate pathognomonic immunohistochemical staining for S-100 and HMB-45, evidence of junctional change with nesting of melanoma cells or spindle cells arranged in fascicles, and invasion of bronchial epithelium in an area without epithelial ulceration. Indeed, other melanotic tumors, such as melanotic medullary carcinoma of thyroid [43] and pigmented neuroendocrine carcinoma should be excluded [44]. In our patient, immunohistochemical staining demonstrated that the tumor cells expressed HMB45, S-100, and MART-1, whereas they did not express CK, EMA, Syn, and high molecular weight cytokeratin. Thus, the diagnosis was reliable.

The precise histogenesis of pulmonary malignant melanoma remains controversial. Most experts support that melanocytes migrate concomitantly with reduced growth of the primordial tubular respiratory tract during fetal growth. It is also suggested that these cases are a metastatic form of an antecedent skin lesion that either is unrecognized or has spontaneously regressed. Interestingly enough, melanocytes and melanocytic proliferation are present in the larynx and esophagus. Furthermore, larynx, esophagus, and lungs all share a common embryologic origin, implying the possible migration of melanocytes [35, 36, 42].

As far as therapy is concerned, patients diagnosed with primary pulmonary malignant melanoma should undergo lobectomy or pneumonectomy with lymph node dissection [2, 42, 45]. Although interferon a-2b (rIFN-*α*-2b) is frequently offered to mucosal melanoma patients as systemic adjuvant therapy, it has not been formally studied in this patient population. Most of the patients with mucosal melanoma have micrometastases at the time of diagnosis of the primary tumor. However, adjuvant therapy has not been studied in a randomized fashion because of the rarity of the disease. The role of chemotherapy is not fully clarified. Biochemotherapy which consists of combination of chemotherapy and immunotherapy is an acceptable choice after always taking into consideration patients' performance status and comorbidities, as well as the relatively expanded toxicity profile of the combination therapy. When aggressive bulky disease is documented, biochemotherapy may be initiated with close monitoring of the patient. Disease stabilization or partial responses have been documented in the cost of toxicity. Our patient did not tolerate well rIFN-*α*-2a combined with DTIC, mandating administration of ipilimumab instead. It seems that overall survival benefit has not been demonstrated in metastatic setting [46]. The reported patient achieved overall survival of 5 months which is in accordance with the literature.

The discovery of activating mutations in the serine/threonine kinase BRAF in approximately 50% of all melanomas led to the Food and Drug Administration (FDA) approval of the BRAF inhibitor vemurafenib in 2011 [47]. Dabrafenib gained FDA approval in 2013, not only for the treatment of BRAF-V600E expressing melanomas, but also for those expressing BRAF-V600K [48]. In the same year, trametinib, the first MEK inhibitor marketed for cancer treatment, was approved by the FDA [49]. Moreover, MEK inhibitors acting in the down signaling RAS pathway can be used preferably in combination with a BRAF inhibitor. Typically, disease remission can be established within weeks, although resistance to therapy is observed usually after a median of 6 months.

For BRAF wild type metastatic melanoma immunotherapy with the anticytotoxic T-lymphocyte antigen-4 (CTLA4) antibody ipilimumab may be the first choice for treatment. Ipilimumab activates the T mediated immune response against the tumor cells which have the ability of immune evasion. Response is achieved late after the therapy, but it can be durable. Finally, in case where c-Kit mutation is present, mainly observed in mucosal or acral melanoma, the BCR-ABL tyrosine kinase inhibitor imatinib has shown some effect but not durable [26, 50]. In our patient BRAF, NRAS, and c-KIT testing was negative for mutations and the multidisciplinary group decided to initiate biochemotherapy.

Recently immunotherapy with programmed death-1 (PD-1) checkpoint inhibitors has raised much attention since both nivolumab and pembrolizumab have been approved by the FDA in 2014 for the treatment of metastatic cutaneous melanoma presenting with excellent risk profile [51]. Even though several new agents have been approved for the treatment of cutaneous melanoma, including the combination of nivolumab and ipilimumab, there is a paucity of published information regarding the efficacy and safety of this combination in other melanoma subtypes. Nivolumab may be effective in mucosal melanoma regardless of the tumor molecular profile, similar to the demonstrated efficacy of nivolumab in cutaneous melanoma regardless of BRAF mutation status. A pooled analysis of data from six clinical studies regarding anti-PD-1 therapy in mucosal melanoma revealed that despite differences in the proportion of patients with tumor PD-L1 expression ≥ 5%, overall response rate was similar between subtypes for nivolumab monotherapy and combination therapy. In contrast, lower activity in mucosal melanoma was observed across treatment groups for patients with tumor PD-L1 expression < 5% [52]. Immunotherapy was not the first choice for our patient due to the long period needed to manifest any disease control. Unfortunately the patient died due to massive pulmonary embolism and the response to the immunotherapy cannot be assessed.

Palliative radiation therapy is utilized when bulky metastatic disease is present. Irradiation of the brain is a choice when multiple brain lesions are evident on brain imaging. This was the case in our patient. WBRT has a palliative intent whereas stereotactic radiosurgery can be applied when a restricted number of metastases are observed in central nervous system [46].

## 4. Conclusion

In this report, we added a new case of primary pulmonary malignant melanoma bringing the total number up to 41 patients in the frame of English literature. Overall, this is the rarest type of visceral melanoma and accurate diagnosis requires detailed investigation and fulfilment of specific criteria. It can appear as either a single pulmonary mass or metastatic disseminated disease. Lobectomy or pneumonectomy with lymph node dissection is the gold standard for local treatment. With the new era in melanoma therapeutics there is optimism for longer periods of survival. Due to the rarity of primary pulmonary malignant melanomas, randomized trials for assessing the relative benefits of different treatment modalities are extremely challenging.

## Figures and Tables

**Table 1:** Cases of primary pulmonary malignant melanomas reported in the literature.

Author, year of publication, reference	Age at diagnosis and sex	Tumor		Treatment		Site of metastasis	Outcome
		Size (cm)	Site	Operative	Adjuvant		
Kunkel and Torrey, 1916 [6]	40 F	2,0	Right hilum	None	None	NA	Died 8 mo after diagnosis
Carlucci and Schleussner, 1942 [7]	48 F	5,0	RLL	Pneumonectomy	None	NA	Died immediately postoperatively
Salm, 1963 [8]	45 M	2,0	LLL	Pneumonectomy	None	RUL	Died 6 mo postoperatively
Reed III and Kent, 1964 [9]	71 M	3,5	LLL	Lobectomy	None	None	Alive 10 yr postoperatively
Reid and Mehta, 1966 [10]	60 F	4,5	RLL	Pneumonectomy	None	None	Alive 11 yr postoperatively
Jensen and Egedorf, 1967 [11]	61 F	10	LUL	Segmental resection	None	Left lung-brain	Died 7 mo postoperatively
Allen and Drash, 1968 [12]	40 F	5,0	RLL	Lobectomy	None	Not reported	Not reported
Taboada et al., 1972 [13]	56 M	4,0	LLL	Lobectomy	None	Left lung, brain, liver	Died 14 mo postoperatively
	40 M	2,5	LUL	Pneumonectomy	None	None	Alive 3 yr postoperatively
Weshler et al., 1980 [14]	62 F	NA	Left main bronchus	Thoracotomy	Radiation	Liver, eye	Died 4 mo postoperatively
Robertson et al., 1980 [15]	70 F	NA	RML to carina	None	Radiation	Liver, ribs, lymph nodes	Died 9 wk after diagnosis
Gephardt, 1981 [16]	47 M	NA	Left main bronchus	None	None	None	Died after being diagnosed
Carstens et al., 1984 [17]	29 F	5,0	RUL	Lobectomy	Chemotherapy	Lungs, liver, heart, bone	Died 1 mo postoperatively
Cagle et al., 1984 [18]	80 M	1,5	Minor fissure	Excisional biopsy	Radiation	Left lung	Died 5,5 mo after diagnosis
Demeter et al., 1987 [19]	56 M	4,0	RUL	Pneumonectomy	Chemotherapy	Heart, left lung	Died 1 mo postoperatively
Alghanem et al., 1987 [20]	42 F	6,0	LLL	Lobectomy	None	None	Alive 2,5 yr postoperatively
Santos et al., 1987 [21]	58 M	5,0	RLL	Lobectomy	None	None	Alive 18 mo postoperatively
Bagwell et al., 1989 [22]	62 M	1,0	LUL	Lobectomy	None	Heart, lymph nodes	Died 2 mo postoperatively
Bertola et al., 1989 [23]	30 F	3,0	LLL	Lobectomy	None	Lungs, heart, brain	Died 5 mo postoperatively
Ost et al., 1999 [24]	90 M	6,0	LUL	None	None	NA	Died at time of diagnosis
Erdal et al., 2000 [25]	59 M	8,0	RLL	Lobectomy	Chemotherapy Adjuvant a-interferon	None	Alive 30 mo postoperatively
Dountsis et al., 2003 [26]	41 F	NA	RUL	Pneumonectomy	Chemotherapy Adjuvant a-interferon	None	Alive 18 mo postoperatively
Reddy et al., 2007 [27]	74 M	8,0	LLL	Lobectomy	Chemotherapy N/A	None	Died 10 mo postoperatively
Zuckermann et al., 2011 [28]	68 F	5,0	RUL	Lobectomy	Chemotherapy Adjuvant a-interferon	None	Alive 6 yr postoperatively
Seitelman et al., 2011 [29]	89 M	4,5	LLL	Lobectomy	None	None	Alive 5 yr postoperatively
Neri et al., 2011 [30]	58 M	2,8	LLL	Lobectomy	Chemotherapy Adjuvant dacarbazine	Left lung	Died 6 mo postoperatively
	69 F	4,0	RUL	Lobectomy	Chemotherapy Adjuvant darbazine	Liver, brain, skin, lung	Died 6 mo postoperatively
Gong et al., 2012 [31]	52 F	2,0–2,4	LUL, LLL	None	Chemotherapy dacarbazine	Brain	Died 4 mo postoperatively
	65 F	6,0	RLL	Lobectomy	Chemotherapy Adjuvant darbazine	None	Alive 6 mo postoperatively
Ouarssani et al., 2012 [32]	68 M	6,0	RLL	None	Chemotherapy dacarbazine, vincristine, nimustine hydrochloride	Left lung	Died 2 mo after diagnosis
Dos Santos et al., 2013 [33]	62 F	5,0	LUL	None	Chemotherapy dacarbazine	Thoracic vertebra-pleural effusion	Alive 12 mo after diagnosis
Kamaleshwaran et al., 2014 [34]	56 M	3,2	LLL	Pneumonectomy	Radiation	Cervical spine	Alive 6 mo after diagnosis
Zhang et al., 2015 [35]	60 M	4,5	LLL	Pneumonectomy	Chemotherapy Adjuvant a-interferon	None	Alive 18 mo postoperatively
Gupta et al., 2015 [36]	58 F	9,0	LUL	None	None	None	Died 2 mo after diagnosis
Postrzech-Adamczyk et al., 2015 [37]	69 F	6,0	RUL	Lobectomy	Chemotherapy Adjuvant dacarbazine	None	Died 6 mo postoperatively
	63 M	5,0	LUL	None	None	None	Died 2 mo after diagnosis
Hwang et al., 2015 [38]	82 F	8,0	RLL	None	None	None	Died 3 mo after diagnosis
Filippini et al., 2015 [39]	55 M	3,0	RUL	None	Dacarbazine with a-interferon	Skin-brain	Died 3 mo after diagnosis
Kim et al., 2016 [40]	69 M	5,0	RUL	None	None	Thoracic vertebra-brain	Died 3 mo after diagnosis
Feng et al., 2016 [41]	60 M	3,0	RUL-LLL	None	None	Lymph nodes	Died 2 mo after diagnosis

LLL, left lower lobe; LUL, left upper lobe; RLL, right lower lobe; RML, right middle lobe; RUL, right upper lobe; NA, not available; mo, months; yr, years.
